# Supplementary material for: Intraneuronal binding of amyloid beta with reelin—Implications for the onset of Alzheimer’s disease
Source: PLoS Comput Biol. 2025 Jan 7;21(1):e1012709. doi: 10.1371/journal.pcbi.1012709 (PMC11741591; doi:10.1371/journal.pcbi.1012709)
Supplement: Attachment — Submitted filename: PLOS_review (1).pdf [file pcbi.1012709.s001.pdf]

Paper Review

This study is a great addition to the growing field of mathematical modelling of neurodegeneration. I really enjoyed the presentation of the problem and the questions the paper aims to address in the introduction. It is very clearly structured. This paper investigates the role of intracellular amyloid beta (Aβ), a peptide known to be a key factor in Alzheimer's disease (AD) development. Recent research shows that in layer II neurons of the entorhinal cortex—crucial for memory formation and expressing high levels of reelin—Aβ binds directly to reelin. These neurons are among the first to die in AD patients. If reelin makes Aβ inert, it could protect neurons from Aβ's detrimental effects. However, this is puzzling since these neurons are major sites for AD onset. Using a mathematical model, the paper offers a possible explanation for this apparent contradiction by proposing that these neurons produce more Aβ than those with lower reelin levels.

Major Corrections

1. **Model Validation:** The authors state (lines 312-321) that Figures 1 and 2 can be validated against wild-type mice data. However, there is no explicit model validation or comparison to data (not necessarily the mouse data) that supports the observations from the model. Please provide citations to demonstrate whether the results align with observations from other studies, that may support your hypotheses to strengthen the validation of your model.

Minor Corrections

1. **Readability:** Some sentences are long and hard to read, such as the sentence: *Considering that numerous studies of the human brain using live imaging, immunohistochemistry and biochemistry, supported by experimental results from rodent and cell models, point to a role for intracellular Aβ in non-fibrillated forms in the onset of Alzheimer's disease, this is an intriguing discovery, as it suggests that reelin can function as a sink for intracellular Aβ.* This is an example of a sentence could be simplified for better readability.

2. **Long-Term Implications of the Model:** The model currently addresses short time periods (approximately five days post-infection), after which amyloid reelin concentrations return to baseline. The paper could discuss how these short-term dynamics contribute to Alzheimer's Disease (AD) development over years. Consider discussing scenarios such as repeated infections or damaged recovery mechanisms that could lead to long-term effects, or is it just through tau seeding?

3. **Choice of variables:** You use τ as a parameter in the model but this coud be a confusing choice as the protein τP is discussed in this field. Consider using a different notation for clarity.

4. **Line 107:** The subsection title *Explanation of the differential equations* seems redundant. It's more effective to directly explain the differential equations without this, but this is optional - the paper is nicely structured.

5. **Line 111:** The word *below* is used to describe what is actually *above.* Please correct this.

6. **Parameter Table:** It would improve readability to include a table of parameter values and units at the start or end of the parameterization section. While the justification is good, the values are somewhat lost in the text.

7. **Line 287:** The statement about being agnostic regarding the sensing mechanisms is repeated unnecessarily. It can be removed.

8. **Line 306:** Please clarify why the value of 44000 was specifically chosen for $\alpha_{\text{infection}}$.

9. **Figure 1:** The colors in the figures need to be clearly explained in the text or caption. For instance, if the orange color represents the infected phase, this should be explicitly mentioned.

10. **Line 361:** A reference is needed for the *in silico* experiments mentioned. These studies seem to be foundational to the Figure 4 analysis, so it's important to cite them.
