# Supplementary material for: Intraneuronal binding of amyloid beta with reelin—Implications for the onset of Alzheimer’s disease
Source: PLoS Comput Biol. 2025 Jan 7;21(1):e1012709. doi: 10.1371/journal.pcbi.1012709 (PMC11741591; doi:10.1371/journal.pcbi.1012709)
Supplement: Attachment — Submitted filename: Response to reviewers.docx [file pcbi.1012709.s002.docx]

**Response to Reviewers**

We wish to thank the reviewers for taking the time and effort to provide us with highly valuable constructive criticism for this manuscript.

Please note that when we resubmitted the paper a few weeks ago we were asked by the Editorial Service of the journal to either delete the phrase ‘data not shown’ regarding our semi-quantitative analysis of reelin level in cortical neurons expressing low levels of reelin or amend the supplementary material to include the referenced data. Because the general presence of reelin in cortical neurons is a critical premise for the whole study, we decided to do a proper quantitative immunohistochemical analysis in wild-type rats. Due to their importance, the results are now presented in a new Figure 1.

In what follows we provide a point-by-point response to the issues raised by each reviewer.

**Response to Reviewer 1**

*Major Corrections*

1. *Model Validation: The authors state (lines 312-321) that Figures 1 and 2 can be validated against wild-type mice data. However, there is no explicit model validation or comparison to data (not necessarily the mouse data) that supports the observations from the model. Please pro- vide citations to demonstrate whether the results align with observations from other studies, that may support your hypotheses to strengthen the validation of your model.*

**Response**: We are not aware of other studies providing data that can be used as a conclusive testbed for our predictions regarding the predictions given in Figs. 1 and 2. Having said that, we would like to emphasize that the COLBOS genotype data arguably qualifies as a testbed for the model. And since the model successfully predicts what is actually observed in humans regarding p-tau formation by just reducing the affinity between *Aβ* and reelin, it indirectly supports the proposed model as such. Likewise, the ApoEε4/ε4 case shows, abeit less convincingly than the COLBOS case, that there may be a constitutive p-tau seeding under baseline conditions, which is consistent with the pronounced neurofibrillary tangle (NFT) pathology observed in this genetic background. Together, these two cases can therefore be regarded as a preliminary validation.

In addition, thanks to both reviewers pushing us on this point, we finally realised that the best available validation data were in fact in-house. The data were published last year in the same paper that documented the interaction between reelin and Aβ (Brain Commun. 2023;5(2), doi:10.1093/braincomms/fcad115). They show the effect of reducing the reelin level in Re^+^alECLII neurons in McGill-R-Thy1-APP homozygous transgenic rats by using an miRNA intervention to lower the intracellular reelin levels, which causes a concomitant lowering of Aβ42 levels relative to control Re^+^alECLII neurons in the same individual. The model is capable of exactly reproducing the reported mean value changes. This implies that the model adequately captures the dynamics between Aβ42 and reelin under steady-state conditions, strongly supporting its main premise, namely that reelin functions as a sink Aβ42. This in turn supports the main conclusion of this study, saying that the maximum production capacity of Aβ42 is markedly higher in Re^+^alECLII neurons than in neurons expressing low levels of reelin to have a comparable immune response.

We have now made a new section titled *Validation of the model*, where the previous section *The effect of genotype on the immune response in Re^+^alECLII neurons* is now the first subsection. The second subsection is titled *Predicting the effect of lowering the level of reelin in Re^+^alECLII neurons* and it describes the new validation test. It includes a new Figure 5 to ease understanding, where the two first panels are extracted from Figure 4A in Brain Commun. 2023;5(2), doi:10.1093/braincomms/fcad115.

*Minor Corrections*

1. *Readability: Some sentences are long and hard to read, such as the sentence: Considering that numerous studies of the human brain using live imaging, immunohistochemistry and biochemistry, supported by exper- imental results from rodent and cell models, point to a role for intracellular Aβ in non-fibrillated forms in the onset of Alzheimer’s disease, this is an intriguing discovery, as it suggests that reelin can function as a sink for intracellular Aβ. This is an example of a sentence could be simplified for better readability.*

**Response**: We have rephrased this particular sentence, and made edits throughout the ms where we have anticipated that the reviewer’s concern might apply. These can be found in the Track changes version.

2. *Long-Term Implications of the Model: The model currently addresses short time periods (approximately five days post-infection), after which amyloid reelin concentrations return to baseline. The paper could discuss how these short-term dynamics contribute to Alzheimer’s Disease (AD) development over years. Consider discussing scenarios such as repeated infections or damaged recovery mechanisms that could lead to long-term effects, or is it just through tau seeding?*

**Response**: Beyond what we wrote under *Concluding remarks*, we deliberately tried to avoid speculations about the long-term effects of repeated dysfuntional activation of this native immune response due to lack of a proper model and the contentiousness of the issue. Since submission of this manuscript we have, however, spent considerable effort on making a model capable of making long-term (> 20 years) predictions consonant with emprical data regarding accumulation of the Aβ_reelin_ complex as well as accumulation of aggregation-prone p-tau fragments due to the recurrent inflammation events characterizing the physiology of the elderly. We are not yet finished with the analysis, and in any case it is so complex that it would confound the main message of this paper. However, to meet the reviewer’s request we have revised and expanded the *Concluding remarks* section to point to possible long-term scenarios which we now believe are supported by a quantitative model that stands upon the shoulders of the model presented in this paper.

3. *Choice of variables: You use τ as a parameter in the model but this coud be a confusing choice as the protein τP is discussed in this field. Consider using a different notation for clarity.*

**Response**: Although the term τP is used by some authors, we do not think the use of the parameter τ to denote the production rate of reelin will cause any confusion since it is clearly defined and the notation for the hyperphosphorylated tau protein (p-tau) is given on the third line in the Introduction. So we would prefer not to change it unless this is deemed absolutely necessary.

4. *Line 107: The subsection title Explanation of the differential equations seems redundant. It’s more effective to directly explain the differential equations without this, but this is optional - the paper is nicely structured.*

**Response**: Yes, this alternative way of presentation is in many cases preferable. However, in this case we decided to keep the equations together so that the mathematically inclined reader could make a fast assessment of the equations and it premises, while readers not so well versed in computational biology but having domain competence could acquire an understanding of the model and its premises from the stand-alone verbal explanation. The majority of researchers in the Alzheimer field is not skilled in mathematical modelling, and this split was done because we think it facilitates a broader readership. Since Reviewer 1 opens for keeping the sections as they are and that Reviewer 2 does not have any concerns regarding this, we would prefer to keep the split as it is.

5. *Line 111: The word below is used to describe what is actually above. Please correct this.*

**Response**: The *below* referred to the explanations of the two equations below, but we agree this was unfortunate. We have removed *below* from the sentence.

6. *Parameter Table: It would improve readability to include a table of parameter values and units at the start or end of the parameterization section. While the justification is good, the values are somewhat lost in the text.*

**Response**: We fully agree, and we have now made a Table 1 providing all parameter values and their units in the two types of neurons studied.

7. *Line 287: The statement about being agnostic regarding the sensing mechanisms is repeated unnecessarily. It can be removed.*

**Response**: We fully agree, and the sentence is deleted.

8. *Line 306: Please clarify why the value of 44000 was specifically chosen for α infection.*

**Response**: It was chosen to achieve an assumed steady state Aβ concentration of 100 nM during the infection phase. We have rephrased the paragraph to make this more clear, and to show that the main message of the paper is not dependent on this being the correct figure.

9. *Figure 1: The colors in the figures need to be clearly explained in the text or caption. For instance, if the orange color represents the infected phase, this should be explicitly mentioned.*

**Response**: Yes, we should have done this. It is now corrected.

10. *Line 361: A reference is needed for the in silico experiments mentioned. These studies seem to be foundational to the Figure 4 analysis, so it’s important to cite them.*

**Response**: This is a misunderstanding, for which we take the full responsibility. We did not refer to another numerical experiment, but just using the term *in silico experiment* to decribe the use of the model to test the effect of changing the genetic background. We have now exchanged the term with *model*.

1. **Response to Reviewer 2**

   1. *Could you please add some mathematical stability analysis for the obtained equilibrium and show other possible steady state equilibrium points (if any)? This additional analysis can be included as supplementary information. This could be important for other research fellows who work with mathematical modelling.*

**Response**: In the revised ms we now include a statement (just after providing the steady state equations) about the existence of a second steady state, but that we do not consider it as three of the four steady state solutions are negative with the parameterization used for the two types of neurons. In addition, we have included a new subsection on stability (*Linear stability analysis*) following the subsection on feedback structure where we introduce the Jacobian. Here we describe how the eigenvalues were found, and that all four eigenvalues are negative real numbers for the two baseline steady states, implying that the steady states are locally stable. We also mention that the same is true for the two steady states associated with the infection phase. We do not think it necessary to provide a more extensive stability analysis given the scope of the paper.

2. Could you please add some data or data analysis to support your results if any? It is not only to validate your model, but also to trigger further experiments because in your work you tried to resolve paradoxical hypotheses.

**Response**: Please see our response to Reviewer 1 regarding major corrections, point 1.

Sincerely,

Asgeir Kobro-Flatmoen
